# Technical considerations when using the EEG export of the SEDLine Root device

**DOI:** 10.1007/s10877-020-00578-9

**Published:** 2020-08-19

**Authors:** Falk von Dincklage, Carlo Jurth, Gerhard Schneider, Paul S García, Matthias Kreuzer

**Affiliations:** 1 Klinik für Anästhesiologie m.S. operative Intensivmedizin (CCM/CVK), Charité – Universitätsmedizin Berlin, Berlin, Germany; 2 Department of Anesthesiology and Intensive Care, Klinikum rechts der Isar, School of Medicine, Technical University of Munich, Munich, Germany; 3 Department of Anesthesiology, Neuroanesthesia Division, Columbia University Medical Center, New York Presbyterian Hospital, New York, USA

**Keywords:** Monitoring, Electroencephalography, Anesthesia general, Patients

## Abstract

Electroencephalographic (EEG) patient monitoring during general anesthesia can help to assess the real-time neurophysiology of unconscious states. Some monitoring systems like the SEDLine Root allow export of the EEG to be used for retrospective analysis. We show that changes made to the SEDLine display during recording affected the recorded EEG. These changes can strongly impact retrospective analysis of EEG signals. Real-time changes of the feed speed in the SEDLine Root device display modifies the sampling rate of the exported EEG. We used a patient as well as a simulated EEG recording to highlight the effects of the display settings on the extracted EEG. Therefore, we changed EEG feed and amplitude resolution on the display in a systematic manner. To visualize the effects of these changes, we present raw EEG segments and the density spectral array of the recording. Changing the display’s amplitude resolution affects the amplitudes. If the amplitude resolution is too fine, the exported EEG contains clipped amplitudes. If the resolution is too coarse, the EEG resolution becomes too low leading to a low-quality signal making frequency analysis impossible. The proportion of clipped or zero-line data caused by the amplitude setting was > 60% in our sedated patient. Changing the display settings results in undocumented changes in EEG amplitude, sampling rate, and signal quality. The occult nature of these changes could make the analysis of data sets difficult if not invalid. We strongly suggest researchers adequately define and keep the EEG display settings to export good quality EEG and to ensure comparability among patients.

**Electronic supplementary material:**

The online version of this article (10.1007/s10877-020-00578-9) contains supplementary material, which is available to authorized users.

## Introduction

Monitoring the brain during general anesthesia by means of electroencephalographic (EEG) recordings can help to assess an unconscious patient, as anesthetic drugs induce changes in neuronal firing that are reflected as specific EEG activity. Most commonly the unconscious brain exhibits a general slowing of the EEG oscillations and an increase in amplitude [1]. Many commercial devices use EEG spectral information to produce a dimensionless index that represents the hypnotic component of surgical anesthesia. The SEDLine Root device, is a brain function monitor (Masimo, Irvine, CA, USA) [2]. It processes frontal EEG information to calculate the patient state index (PSI) that attempts to mark the patient’s level of arousal. The device also has an automated method for burst suppression detection. The device provides an export function that allows data collection and retrospective analysis of the recorded EEG. The data can be stored in the European data file format (EDF) [3] on an USB stick. Possession of these EEG recordings in an easily readable format provides the opportunity to conduct research on EEG features that occur during general anesthesia. Earlier versions of the SEDline monitor were used to detect burst suppression EEG and describe alpha oscillatory activity as a target for anesthesia maintenance [4]. In this manuscript, we describe how intraoperative modification of the display features can not only influence the displayed EEG but also the recorded EEG file when stored in the EDF file format. These difficulties include undocumented changes in EEG sampling rate and amplitude as well as data “clipping” (EEG amplitudes beyond the y-axis limits). These changes can lead to corrupted EEG files and perhaps falsify the results from subsequent analysis.

## Methods

For our investigation we used EEG recordings obtained from a sedated intensive care patient that was included in the clinical trial “*Validation of methods for monitoring nociception and pain prediction in the ICU*”. This study was performed in accordance with the ethical standards as laid down in the 1964 Declaration of Helsinki and its later amendments. It was approved and the requirement for written informed consent was waived by the Institutional Research Ethics Committee of Charité - Universitätsmedizin Berlin (vote number: EA1/151/16). The trial was registered prior to patient enrollment at the German Clinical Trial Register (DRKS00011206; Principal investigator: Dr. Falk von Dincklage, Date of registration: 11.01.2017).

### SEDLine EEG recording

When using the SEDLine, an electrode sensor is attached to the patient’s forehead. The sensor consists of six electrodes that record brain electrical activity from recording positions Fp1, Fp2, F7 and F8 according to the international 10–20 system with the reference and ground electrode placed on the center of the forehead (around Fz). The SEDLine monitor processes the EEG information to calculate the PSI. It also displays the changes in spectral EEG power over time as a density spectral array (DSA) on the monitor’s display to provide the anesthesiologist with additional information regarding the oscillatory composition of the EEG. Further, the raw EEG can also be displayed on the monitor. In order to optimally visualize the EEG trace, the anesthesiologist can adjust the time-scale of the EEG as well as the amplitude resolution. The scaling is either 15 mm or 30 mm per second and the amplitudes can be adjusted to 1, 2, 3, 5, 10, 25, 50, or 100 µV per mm.

### SEDLine EEG export

It is possible to export a recorded EEG file from the SEDLine Root device in European Data Format (EDF file, .*edf* extension).Using the export function, the EEG can be exported to a USB-drive as an EDF file.

The EDF files consist of a header record and a data record. The data record contains the time series data, i.e., the amplitude values recorded at equally-spaced time points. In exported SEDLine EEG files the data record contains numeric information from the four frontal EEG channels. The header record contains information regarding electrode positions, patient and time information etc. The sample rate is not given explicitly, but can be obtained by dividing the value of the header variable ‘*samples*’ by the value of the header variable ‘*duration*’. Hence, one sample rate value can be derived that reflects the sample rate for the entire recording. The sample rate is defined at the beginning of the recording.

### Change of EEG display protocol

#### Clinical EEG recording

We suspected that the choice of parameter settings on the SEDLine display during EEG recording may influence the raw EEG that can be exported to the EDF file. Thus, we investigated this possible effect using EEG recordings obtained from a sedated intensive care patient. The patient used for EEG measurement presented the following characteristic: male, 55 years of age, light analgosedation using propofol, clonidine as well as methyl-lorazepam in the post-operative setting. Because we were only interested in the question of how the monitor settings influence the exported EEG, one patient was considered sufficient. We decided on a patient under propofol-induced sedation because (i) the EEG is consistent during constant low/moderate infusion doses, (ii) propofol causes prominent EEG alpha band activity [5], and (iii) there is no EEG contamination by surgical artifacts in this post-operative patient.

We started the EEG recording with the 30 mm/s time-scale. We then changed the time-scale to 15 mm/s and started our stepwise protocol with an initial amplitude setting of 1 µV /mm. We changed the amplitude resolution, approximately every two minutes, in a stepwise fashion (µV/mm in eight steps;1→2→3→5→10→25→50→100), while keeping the feed constant at 15 mm/s. After completing the eight steps we changed the feed to 30 mm/s and reversed the amplitude setting from 100 µV/mm to 1 µV/mm in the stepwise fashion described before for another eight steps.

**Fig. 1:**
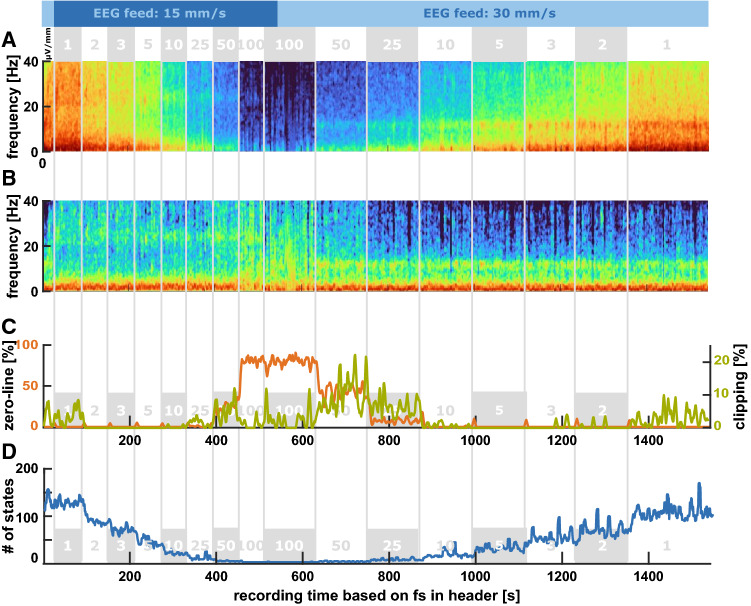
Density spectral arrays (DSA) of the (relative) power spectral density (PSD) for the different display settings depicted above the DSA as well as the amount of clipping and zero-lines in these episodes **a** DSA of the spectral power: Depending on the amplitude setting the recorded EEG amplitudes, i.e., the colors in the DSA change. The width of the ‘unchanged’ settings relates to the EEG feed **b** DSA indicating the relative power: Using the relative power can correct for the change in EEG amplitude, but the influence of the EEG feed still remains **c** Amount of clipping (green) and zero-lines (orange): the amount of clipped EEG amplitudes is substantial for the 1 µV/mm setting as well as for the 50 and 25 µV/mm setting during the 30 mm/s feed. There the clipping is caused by the low resolution of the signal that also causes the high fraction of zero-lines during the 100 µV/mm setting. **d** The number of states, i.e., the number of different amplitude values in the EEG recording decreased with an increase of the EEG amplitude setting. (The trend was smoothed with a moving mean of 5 points)

This stepwise protocol is also presented in Fig. 1. We then exported this recording as an EDF file and analyzed the EEG with MATLAB R2017b (The MathWorks, Natick, MA, USA). We based our analyses on the sample rate indicated in the header of the EDF file, which in our case was 178 Hz, because we started with the 30 mm/s feed. For the analyses, we used channel L1, i.e., the signal recorded from position Fp1.

#### Simulated EEG recording

We further replayed a simulated EEG trace to the SEDLine using an EEG player [6] to channel L1 (Fp1) and channel L2 (F7). Therefore, we generated a white noise signal using the MATLAB *rand* function and filtered the signal to the 9–11 Hz range using the MATLAB *filtfilt* function. We then conducted a stepwise replay of the signal starting with a 30 mm/s feed (i.e., a f_s_=178 Hz) and a 1 µV/mm amplitude resolution. We increased the amplitude setting every 30 s, changed to a feed of 15 mm/s after 30 s in the 100 µV/mm setting. Then we decreased the amplitude resolution again in the same manner. For the analyses, we used channel L1, i.e., the signal recorded from position Fp1. For the evaluation of clipping, we used the signals from Fp1 and F7 in order to evaluate differences.

### Analysis of the EEG stored in the EDF file

In order to examine possible changes in the EEG recording induced by changes in the display settings we calculated the power spectral density (PSD) of the EEG for overlapping EEG segments of 5 s length with a shift of 1 s. Therefore, we used the MATLAB *pwelch* function with default settings and a NFFT = 512. We also calculated the normalized PSD (nPSD) by dividing the PSD for each 5 s EEG episode by the sum of power in the 0.4 to 30 Hz range. We also used a custom MATLAB function to evaluate the amount of clipping in the recorded EEG for each feed and amplitude display setting. Clipping is a signal distortion that limits measures of the EEG amplitude to a specific maximum value, i.e., it causes horizontal EEG traces. This will interfere with frequency analysis. For our analyses we defined a rather conservative clipping setting by only considering a signal clipped, if it showed the horizontal line for longer than 10 data points. We present the results from the (n)PSD analysis as DSA heatmaps.

## Results

### Changes in the amplitude resolution for EEG display affect the stored EEG amplitude

When calculating the PSD from the EEG recording our PSD resolution was 0.35 Hz. The DSA in Fig. 1a shows a change in PSD with every change in the amplitude resolution display setting. The finer the amplitude resolution the higher the power, depicted by the warmer colors in Fig. 1a. We could correct for the differences in power by using the nPSD approach as displayed in Fig. 1b.

**Fig. 2:**
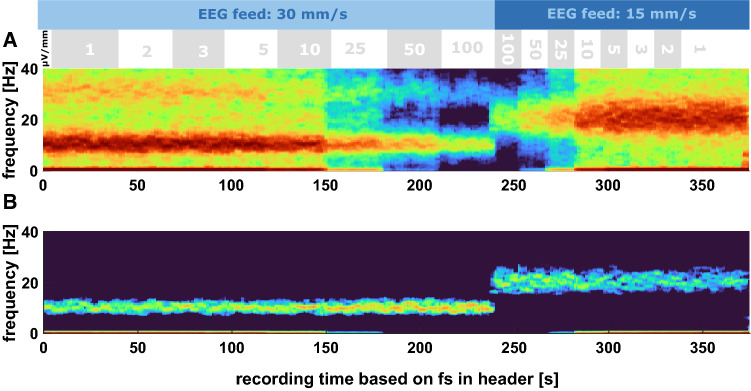
Density spectral arrays (DSA) of the (relative) power spectral density (PSD) for the different display settings depicted above the DSA for the simulated 9–11 Hz oscillatory activity. **a** DSA of the spectral power: Depending on the amplitude setting the recorded EEG amplitudes, i.e., the colors in the DSA change. **b** DSA indicating the relative power: The sudden jump in the dominant frequency range of 9–11 Hz because of the change in the feed from 30 mm/s to 15 mm/s is clearly identifiable

**Fig. 3:**
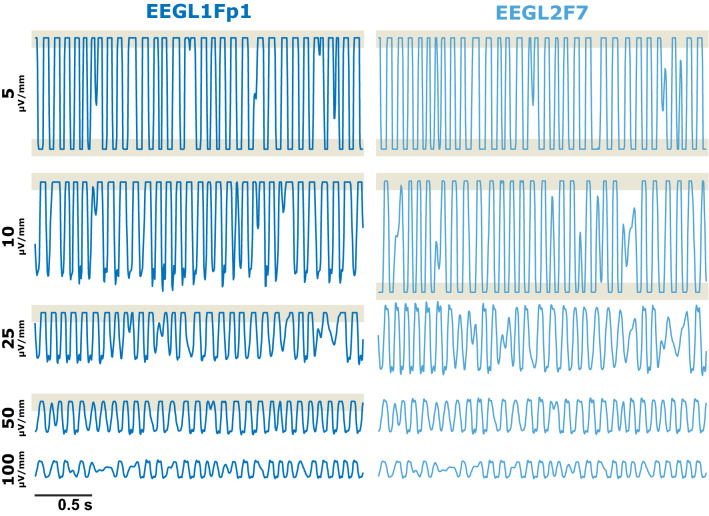
Snippets of the recorded traces from the replay simulated 9–11 Hz oscillatory activity for the different amplitude settings on the display and for the different electrodes Fp1 (left) and F7 (right). The lower the resolution, the less the trace is affected by clipping. The amount of clipping is dependent on the channel position in the display. Because the Fp1 trace is presented in the 1st row of the display this trace is affected by upper clipping for more settings than the F7 trace placed in the center of the SEDLine display

The analyses of the simulated EEG confirmed the influence of the amplitude setting on the display settings as presented in the DSA plot in Fig. 2a. The signal recorded from the SEDLine with the different amplitude settings during the 30 mm/s setting replay shows the difference in recorded signal amplitude as well (Fig. 3). The jump in the DSA occurs because the sampling rate changes without being documented. If you then calculate the DSA with a set frequency resolution, i.e., a defined number of NFFT (number of discrete Fourier transformation) points like 512 in our case, the frequency resolution will be twice as fine in case you change from the initial sampling rate of 178 to 89 Hz.

### Changes in the feed for EEG display affect sample rate of the stored EEG

Further, the wider corridors of unchanged power starting at step 9, i.e., when we changed the feed of the EEG to 30 mm/s, indicate an influence of the feed setting on the sample rate. Another indicator is the change of the dominant oscillatory component around 22 Hz in steps 1 to 8 that got shifted to around 11 Hz in step 9 to 16. When using the DSA derived from the nPSD, this shift is even more obvious. Because the recording was started with 30 mm/s and changed to 15 mm/s before starting the protocol, the dominant ~ 10 Hz oscillatory activity observed in the beginning in a patient under general anesthesia/sedation was shifted to ~ 20 Hz. The change in sample rate is also reflected in an inaccuracy in calculating the length of the recording. When dividing the number of recorded data points per EEG channel by the indicated sample rate we found recording time of around 25 min which does not match the real recording time of around 33 min consisting of 16 steps a 2 min and sometime before and after the protocolled steps. When using the nPSD the jump of the power peak from ~ 10 to ~ 20 Hz as a consequence of changing the feed from 30 to 15 mm/s (Fig. 2b). Also the width of the 9–11 Hz frequency band with strong power in Fig. 2a and b becomes wider, because of the finer frequency resolution, due to the reduced sample rate but keeping the same Discrete Fourier Transformation settings applied by the *pwelch* command.

### EEG distortions: Clipping and low resolution

**Fig. 4:**
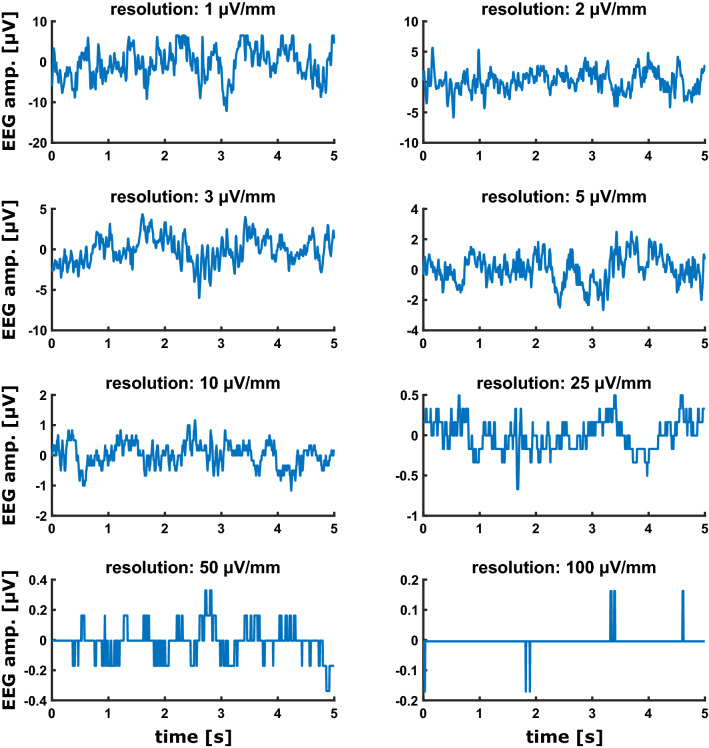
Five second EEG samples recorded during the different EEG amplitude settings on the SEDLine display. The coarser the amplitude scaling on the display, the more stair-like the recorded EEG becomes

Depending on the amplitude resolution the recorded EEG is differently affected by the clipping distortion. The higher the amplitude resolution on the display, the higher the amount of clipping as displayed in Fig. 1c. Especially the 1 and 2 µV/mm settings can lead to a considerable amount of clipped EEG as detected with our 10-point setting for the clipping function. For the low amplitude resolution settings of 50 and 100 µV/mm we also observed horizontal EEG that was not caused by clipping, but by a very low resolution of the recorded EEG that led to a stair-like signal. Figure 4 presents 5 s examples of EEG raw traces (from the second 8 blocks). The number of possible amplitude values as depicted in Fig. 1d “number of state” graph strongly decreased when the amplitude resolution on the display was decreased, i.e. the signal starts to look “rectangular” as in Fig. 4d. But the amount of clipping seems to depend on the channel and the position of the signal on the screen. Figure 3 that displays the recorded simulated data at different amplitude resolutions revealed that in channel L1, clipping occurs also in the amplitude settings 25 and 50 µV/mm, but it does not in channel L2. But in channel L2 we observed a lower-bound clipping in the 10 µV/mm setting. This means that the signal recorded reflects the signal displayed on the screen. L1 is displayed as first of the four EEG traces and is hence influenced by the upper-bound clipping. L2 is the third trace and at the 10 µV/mm the signal goes out of the display and hence becomes clipped. The screenshots in the supplemental Fig. S1 should clarify this issue.

## Discussion

Using the information provided by EEG recordings is recommended to monitor the patient when undergoing a surgical intervention requiring general anesthesia [7, 8]. Processed EEG indices like the BiSpectral index [9] or the Patient State Index [2] attempt to estimate the hypnotic state of a patient as a dimensionless number. For a more reliable assessment of the patient’s state, additional inspection of the raw EEG or the spectrogram is strongly suggested [10]. Further, in order to understand the processed EEG indices in more detail and to search for EEG features that can help to improve the reliability of these indices, the recording of intraoperative EEG is necessary. The SEDLine device allows for export of raw EEG via an USB port. This export function could provide a very useful tool to get EEG recordings to conduct research on. Unfortunately, as described by our results, the exported EEG recordings are dependent on the EEG setting for display on the monitor.

(A) A change in display feed causes a (undocumented) change in sample rate of the recorded EEG.

(B) A change in display amplitude resolution causes a (undocumented) change in the amplitude and affects the quantization of the recorded EEG.

(C) Inadequate amplitude resolution can lead to distortion of the EEG signal (clipping or stair-steps).

(D) The amount of clipping is dependent on the position of the displayed signal on the screen.

When the anesthesiologist starts a recording, the actual sample rate that is dependent on the EEG feed on the display is stored. This sample rate is then denoted in the header section of the EDF file after EEG export. Any changes made to the feed on the display will cause a change in the sample rate at which the EEG is recorded. However, these changes are not logged in the file. Hence, when analyzing the EEG from the EDF file, undocumented changes in the feed (sample rate) can lead to erroneous results. As displayed in Fig. 1 for patient data and Fig. 2 for simulated data, a change in the feed from 30 to 15 mm/s causes a shift of the oscillatory alpha activity presented as a yellow to red band around 10 Hz in the spectrogram to a band around 20 Hz. If the focus is on investigating alpha-band properties, the result would be wrong, because the researcher would analyze the wrong frequencies. In order to check the raw EEG from the EDF file for these “change in feed” events, the duration of the recording should be checked for each case and compared to the proposed length of the recording, i.e., the number of data points in the EDF file divided by the sample rate denoted in the header. If these durations are off, a feed change probably happened. Further, the DSA can be checked. If there are jumps of a dominant band, like for instance alpha under general anesthesia, to either twice or half the frequency, a feed change can be assumed.

Another issue presents the change in the EEG amplitude as well as the quantization of the EEG when recorded at different amplitude resolutions on the display. A pure change in the EEG amplitude could be corrected, for instance, by normalizing the EEG amplitude by means of standard scoring, i.e., the presentation of the amplitude not as (µ)V but as standard deviations, or as normalized PSD as presented in Figs. 1b and 2b. But the choice of amplitude resolution also affects the quantization of the signal. The coarser the amplitude resolution on the display, the more stair-like the recorded EEG (Fig. 4) can be. This means that with lower display resolution, the number of recorded EEG amplitudes values can become lower. Lower quantization leads to an increased amount of white noise content in the spectrum [11]. For the spectral power of the EEG this means an increasing influence of the higher frequencies to the total power which is identifiable as warmer colors at 20 Hz and above in the 100 and 50 µV settings for the patient EEG (Fig. 1b). While the quantization causes the reduction in the resolution and leads to an increased amount of zero-lines, the very fine resolution settings on the display like 1 or 2 µV/mm for the patient EEG or from 1 µV/mm to 10 µV/mm (or even 25 µV/mm in the Fp1 channel) for the simulated data can lead to another problem: clipping. EEG clipping occurs if the amplitude goes into saturation and the maximal (or minimal) possible amplitude values are reached. In the case of the SEDLine we observed hard clipping, i.e., a horizontal line in the time series once the EEG amplitude reached the threshold. Normally, with well-adjusted amplifier settings, clipping occurs during large amplitude artifacts with amplitudes considerably larger than the useful signal like the EEG. In our case presented, clipping affected the artifact-free EEG. Clipped signals cannot be completely recovered, although by assuming an underlying model structure the missing parts can be estimated [12]. Hence, clipping can affect the analytical results. For instance, the (power) spectrum changes with clipping [13]. In order to avoid the quantization as well as the clipping issue the 3 and 5 µV/mm display setting were best suited in the presented EEG measurement. But the 3 and 5 µV/mm setting would not work for the simulated data because of strong clipping. So the display has to be carefully adjusted and each change, if necessary, has to be noted.

In summary, we here investigated and described the impact of the SEDLine display settings on the EEG traces that can be extracted from the USB port of the device in the EDF format. The recorded EEG presents the traces as they are displayed on the screen. If the SEDLine is used for collecting EEG that will be analyzed for scientific questions the display settings should not be changed throughout data collection. Further, the amplitude resolution on the display should be set to a factor that allows collection of clipping free data at a high resolution.

## Electronic supplementary material

Below is the link to the electronic supplementary material.

Electronic supplementary material 1 (PDF 6384 kb)
