# Supplementary figures and images for: Technical considerations when using the EEG export of the SEDLine Root device
Source: J Clin Monit Comput. 2020 Aug 19;35(5):1047–54. doi: 10.1007/s10877-020-00578-9 (PMC8497458; doi:10.1007/s10877-020-00578-9)

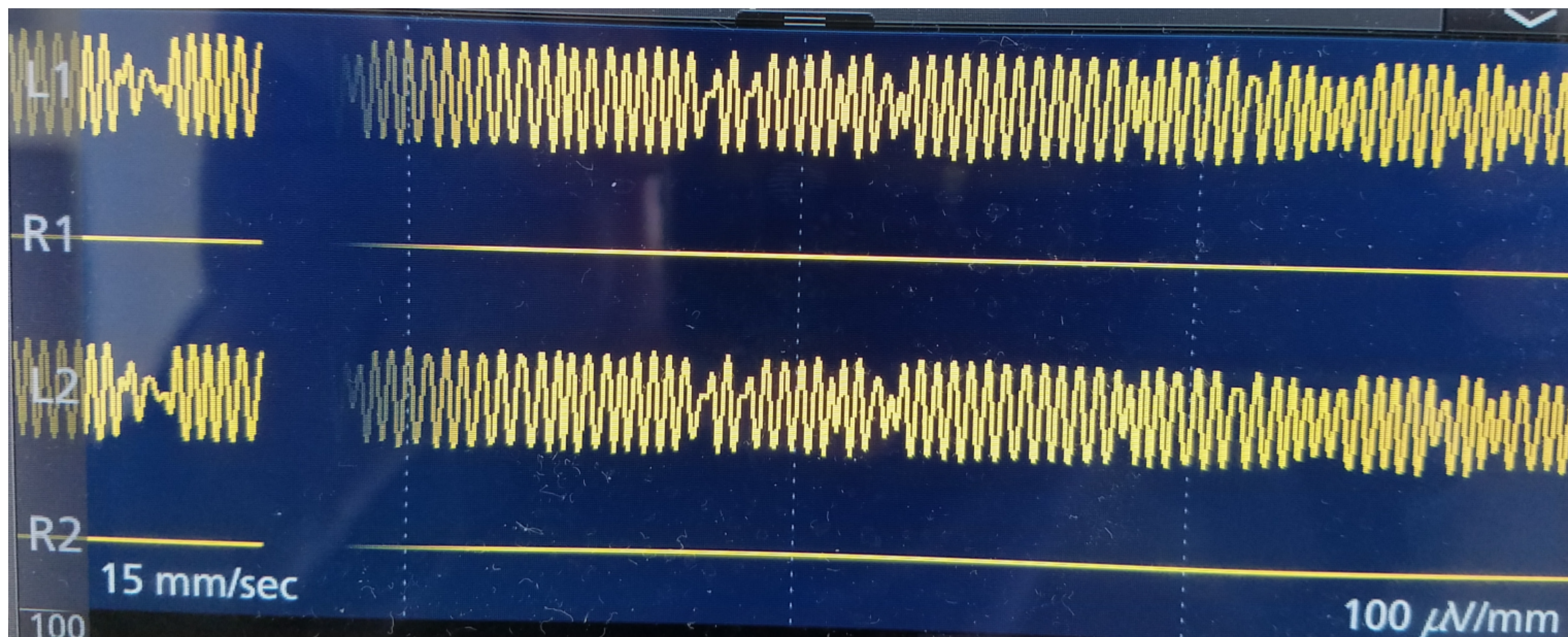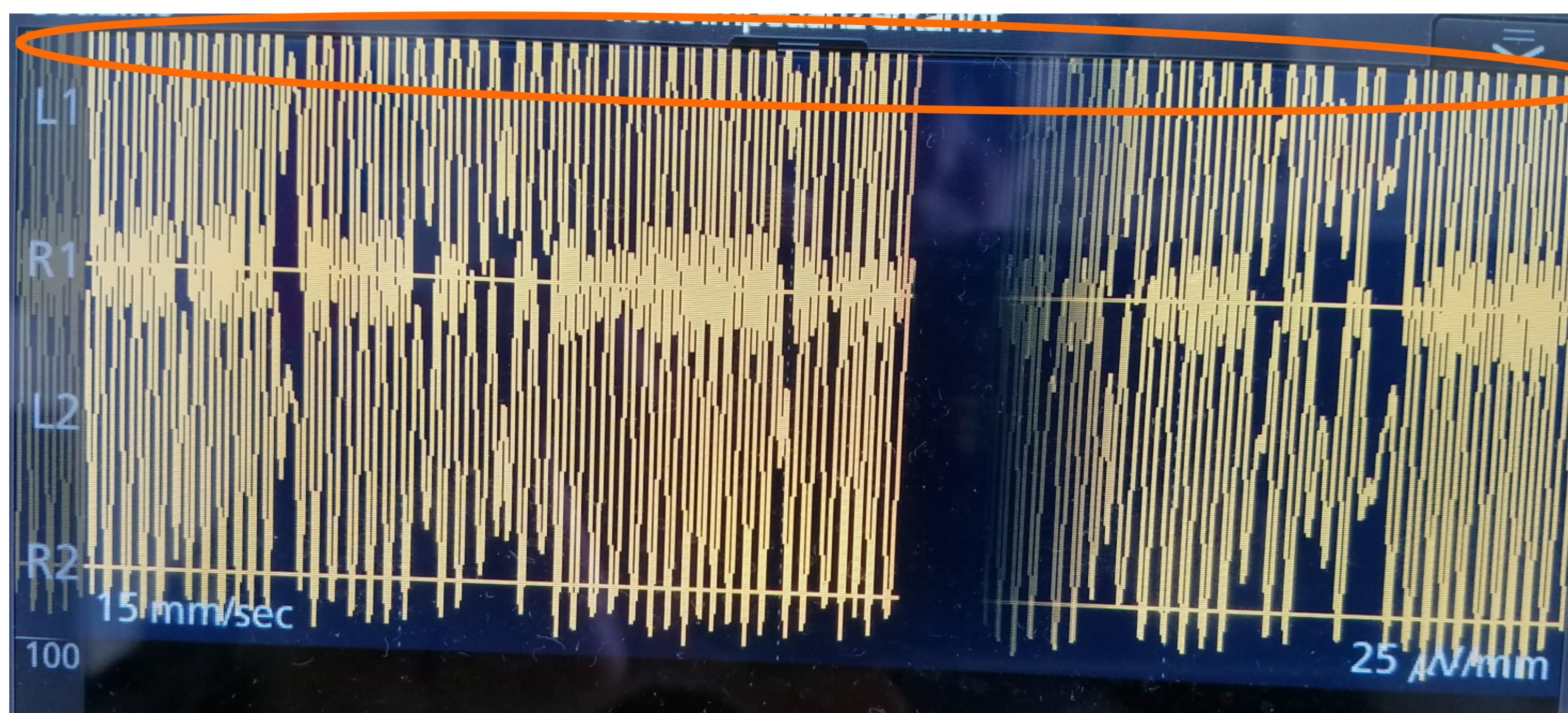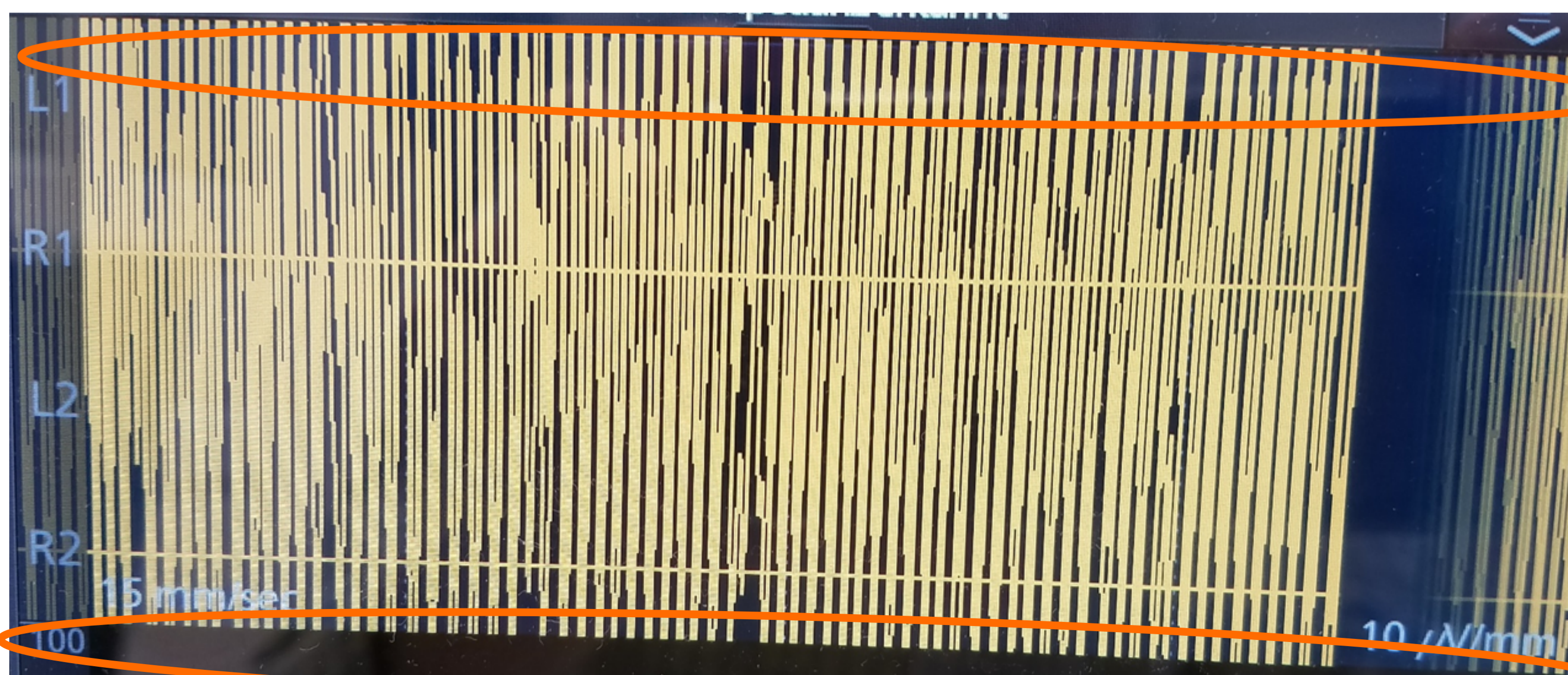

clipping

Supplement: Supplementary file 1 — Electronic supplementary material 1 (PDF 6384 kb) [file 10877_2020_578_MOESM1_ESM.pdf]
